# Rotation matters: CT and MRI yield different tibiofemoral rotation angles in patellofemoral patients. An investigational plateau‐anchored MRI method is descriptively associated with a smaller mean CT–MRI difference

**DOI:** 10.1002/jeo2.70708

**Published:** 2026-04-07

**Authors:** Giuseppe Anzillotti, Marc A. Tompkins, Julie Agel, Spencer M. Comfort, Elizabeth A. Arendt

**Affiliations:** ^1^ Department of Biomedical Sciences Humanitas University Pieve Emanuele, Milan Italy; ^2^ Department of Orthopedic Surgery University of Minnesota Minneapolis Minnesota USA; ^3^ TRIA Orthopaedic Center Bloomington Minnesota USA

**Keywords:** CT, knee osteotomy, MRI, patellofemoral, through knee rotation

## Abstract

**Purpose:**

To compare tibiofemoral rotation (TFR) measured on magnetic resonance imaging (MRI) and computed tomography (CT) in patellofemoral (PF) patients and descriptively report mean differences using an alternative plateau‐anchored MRI measurement method.

**Methods:**

Surgical candidates for tibial derotational osteotomy with both CT and MRI were retrospectively reviewed. Demographics/surgical indication/tibial torsion were recorded. TFR was measured on CT/MRI using standard posterior condylar–posterior tibial axis technique. A plateau‐anchored MRI method utilizes the angle between the distal femoral posterior condylar axis and the axis connecting the proximal tibial plateau's most medial and lateral aspects. Paired *t* tests compared TFR between modalities and MRI methods; subgroup analyses evaluated diagnosis. Pearson correlation assessed association between CT‐based TFR and tibial torsion.

**Results:**

Fifty‐eight knees/46 patients (50 female; mean age 21 ± 7 years; body mass index [BMI] 25 ± 6 kg/m^2^) were analysed. Indications: PF instability ± pain: 20 knees, pain without instability: 38 knees. Mean MRI TFR: 2.11°; CT TFR 8.28°; plateau‐anchored MRI method 9.80°. Mean difference between standard MRI and CT was −6.18° (95% confidence interval [CI] −7.48 to −4.88; *p* < 0.001); the difference between plateau‐anchored MRI and CT was 1.51° (95% CI 0.01–3.01; *p* = 0.048). Bland‐Altman plots showed good inter‐ and intra‐observer agreement. In exploratory subgroup analyses (PF instability ± pain vs. pain without instability), no statistically significant differences were observed in the CT–MRI mean TFR differences. Tibial torsion (38.9° ± 7.0°) did not correlate significantly with CT‐based TFR (*r* = 0.25; *p* = 0.06).

**Conclusion:**

CT and MRI yield systematically different TFR values in the same PF patients; standard MRI yielding lower values than CT. A plateau‐anchored MRI method yielded a smaller mean difference relative to CT than the standard MRI method in this cohort. These results should not be interpreted as demonstrating interchangeability, accuracy, or clinical readiness of the plateau‐anchored method.

**Level of Evidence:**

Level II.

**Abbreviations**
CT: computed tomography
MRI: magnetic resonance imaging
PF: patellofemoral
TFR: tibiofemoral rotation

## INTRODUCTION

Patellofemoral (PF) disorders are characterized by variability in clinical and radiographic presentation [4, 13, 19]. Pain, instability, and or other structural problems can be present in combination or in isolation, offering clinical challenges in diagnosis and treatment [7]. Axial alignment of the limb is one of the risk factors for PF disorders. Rotational abnormalities of the femur and/or tibia may increase the forces acting on the patella and can contribute to patellar maltracking [24], as well as other PF disorders [10]. Limb rotational abnormalities also affect the tibiofemoral joint.

Tibiofemoral rotation (TFR) (or through‐knee rotation) angle is a measurement comparing the posterior femoral condylar axis and proximal tibial posterior condylar axis. It has been variably termed through‐knee rotation, knee version as well as TFR. This measurement is positive if the tibia is externally rotated compared to the femur [11, 24]. Its role in patellar maltracking has recently been investigated to better understand its contribution to PF disorders, in particular recurrent lateral patellar dislocation [2, 16, 20]. A cadaveric biomechanical study demonstrated that TFR angle was correlated with patellar lateral shift distance [9]. Additionally, several clinical studies found that the TFR was significantly higher in patients with recurrent patellar dislocation (RPD) compared to a control group [5, 22, 23]. TFR is hypothesized to influence lateral patellar dislocation, as external tibial rotation relative to the distal femur can lead to relative lateralization of the tibial tubercle, increased lateral tilt, increased medial soft tissue laxity, and modified force vectors, all of which can contribute to patellar instability [11]. Non‐pathologic values were previously registered across a few studies, ranging between −3.8° [11] to −5.7° ± 4.3° [5] on magnetic resonance imaging (MRI), and 9° ± 4° [24], 5.4° ± 5.7° [22] or 4.0° ± 3.7° [23] when assessed through computed tomography (CT) scans. While both CT and MRI have been used, no universal gold standard for TFR measurement exists.

Recently, Jud et al. directly compared TFR measured on CT and MRI in patients with PF instability and found systematically lower MRI‐based values, with the CT–MRI discrepancy moderately correlated with differences in knee flexion angle at acquisition [8]. However, two clinically relevant gaps remain: (1) whether these modality effects generalize to surgical PF cohorts that include pain without instability and (2) whether an MRI approach anchored to tibial plateau morphology yields different mean TFR values than the standard MRI tibial reference.

The primary aim was to compare mean TFR values measured on CT and MRI in surgical candidates for tibial derotational osteotomy with PF disorders. A secondary, exploratory aim was to descriptively compare mean CT–MRI differences obtained using a plateau‐anchored MRI tibial reference versus the standard MRI tibial reference. A further exploratory aim was to evaluate whether segmental tibial torsion is associated with CT‐based TFR [21]. It was hypothesized that standard MRI‐based mean TFR values would differ from CT‐based mean TFR values in this cohort.

Using a PICOT [18] framework: In surgical candidates for tibial derotational osteotomy with PF disorders (P), does MRI‐based TFR measured using the standard method or a plateau‐anchored tibial reference (I) differ from CT‐based TFR (C), as quantified by the paired mean difference in degrees (O), in a retrospective paired‐imaging cohort (T)?

## MATERIALS AND METHODS

### Study design and patients

A retrospective observational cohort study was performed. Approval was granted by the University of Minnesota, Minneapolis institutional review board (IRB# STUDY00020032) to conduct a review of preexisting data from patient charts and imaging acquisitions.

Consecutive surgical candidates for tibial derotational osteotomy (June 2016 to June 2023) were reviewed. Inclusion criteria were: (1) surgical candidacy for tibial derotational osteotomy for PF disorders; and (2) availability of both CT and MRI in the institutional imaging system suitable for TFR measurement. Exclusion criteria were: (1) missing CT or MRI data, and (2) insufficient image quality or slice coverage precluding identification of the required landmarks. Indication was recorded as PF instability ± pain or pain without PF instability.

CT was obtained as part of the preoperative rotational profile evaluation for suspected torsional malalignment and surgical planning for tibial derotational osteotomy. MRI was obtained to evaluate intra‐articular and soft‐tissue pathology and to characterize PF anatomic risk factors used in surgical decision‐making. All patients had CT scans performed at the same institution with the same protocol and read by one of two radiologists. MRIs were performed at a variety of locations and not on the same day. Demographic characteristics and indications for surgery were collected.

### Measurements

**Figure 1:**
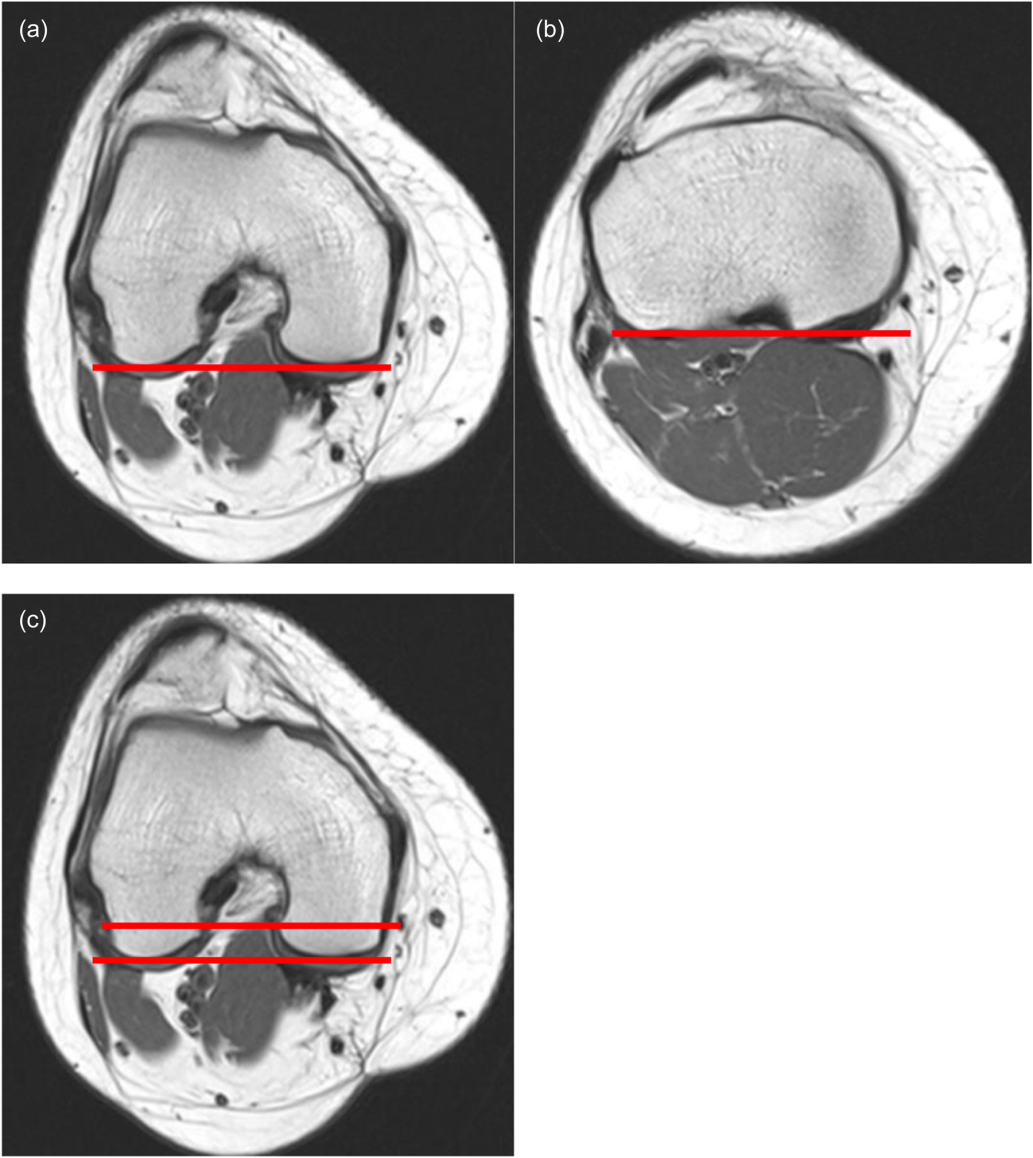
The figure shows the measurement of the TFR angle on MRI, right knee. (a) The posterior femoral condylar line is drawn at the level of the widest anterior–posterior diameter of the condyles, in the closest slice where both posterior condylar cartilages could be appreciated. (b) The posterior tibial condylar line is drawn at the first slice below the menisci, at the level of the posterior cruciate ligament insertion. (c) The two images were superimposed. The TFR angle was calculated as the angle between the two lines (in this example 0°). MRI, magnetic resonance imaging; TFR, tibiofemoral rotation.

**Figure 2:**
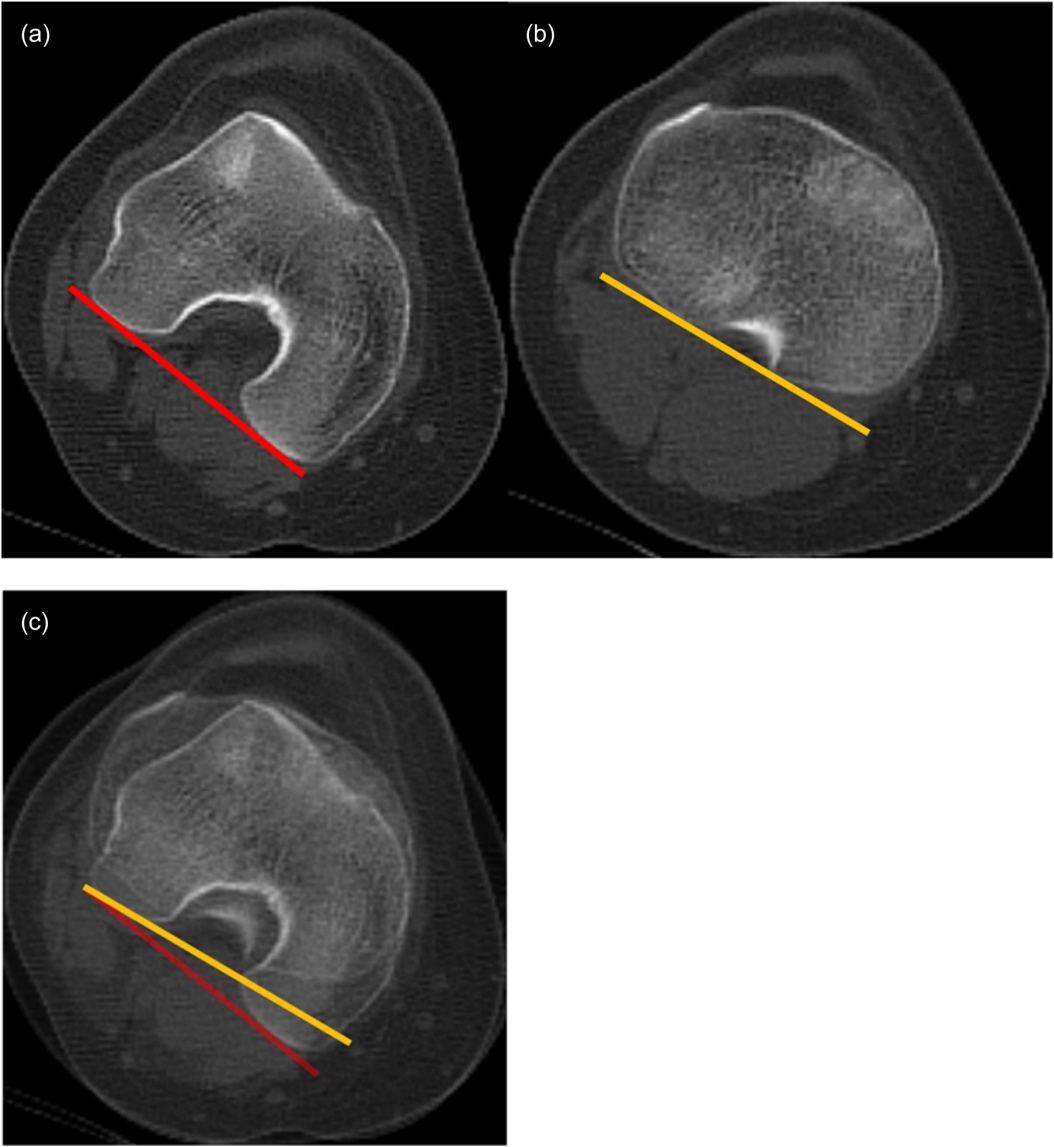
The figure shows the measurement of the TFR angle on CT, right knee, same patient as in Figure 1. (a) The posterior femoral condylar line (red) is drawn at the level of the widest anterior–posterior diameter of the condyles, in the closest slice where both posterior condylar cartilages could be appreciated. (b) The posterior tibial condylar line (yellow) is drawn at the first slice below the menisci, at the level of the posterior cruciate ligament insertion. (c) The two images were superimposed. The TFR angle was calculated as the angle between the two lines (in this example 9°; the tibia is externally rotated in relation to the femur). CT, computed tomography; TFR, tibiofemoral rotation.

TFR angle was measured with the standard published technique on both MRIs (Figure 1) [5, 11] and CTs (Figure 2) [22, 23, 24], both using the angle between the posterior femur condylar line and posterior tibial plateau line.

**Figure 3:**
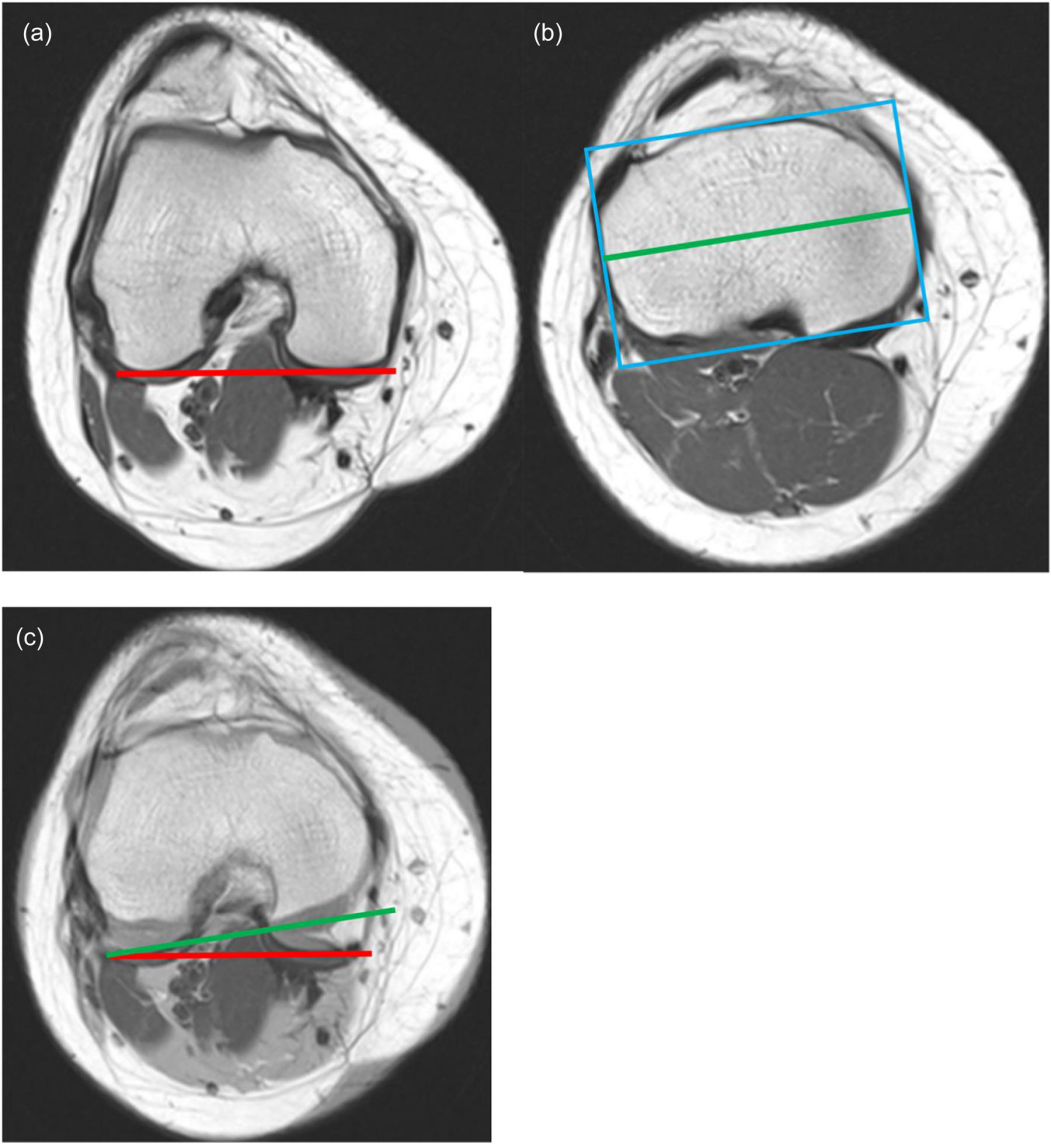
Plateau‐anchored MRI TFR method, right knee (same patient as Figures 1 and 2). (a) Femoral posterior condylar line at the slice of maximal condylar AP diameter. (b) On the first tibial slice below the menisci at the PCL tibial insertion, a minimum‐area bounding rectangle is placed snugly around the tibial plateau without subsequent manual rotation; the tibial ML axis is the line connecting the midpoints of the rectangle's short sides. (c) Superimposition of femoral and tibial lines; TFR is the acute angle between them (positive for tibial external rotation). Example: 10°. AP, anteroposterior; ML, medial–lateral; MRI, magnetic resonance imaging; PCL, posterior cruciate ligament; TFR, tibiofemoral rotation.

An alternate measuring method on MRI was created by the authors, which utilizes the angle between the posterior condylar axis of the distal femur and the axis connecting the widest point of the tibial plateau below the menisci, as described in Figure 3. In detail:

‐ *Slice selection*: Use the first axial slice immediately below the meniscal undersurface at the level of the posterior cruciate ligament (PCL) tibial insertion (same level used for the tibial line in the standard method). Do not rotate the DICOM image; preserve scanner orientation.

‐ *Anatomic boundary*: Delineate the outer cortical rim of the tibial plateau. Ignore meniscal tissue and osteophytes; if osteophytes are present, trace the apparent native cortical contour.

‐ *Rectangle placement*: Place a minimum‐area bounding rectangle tightly around the cortical rim of the plateau on that slice (visually, the rectangle's sides are tangent to the anterior, posterior, medial and lateral cortical contours). Its orientation is defined by the tightest fit to the plateau cortex.

‐ *Axis definition (angle line on tibia)*: The tibial plateau medial–lateral (ML) axis is the line connecting the midpoints of the two short sides of the rectangle.

‐ *Femoral line*: On the femoral slice (the first axial slice starting from the joint level at the widest anteroposterior (AP) diameter where both posterior femoral condylar cartilages are visible), draw the posterior condylar line per the standard technique.

‐ *Image fusion*: Superimpose the femoral line and the tibial ML axis, as in the standard method.

‐ *Angle setting and sign convention*: Measure the acute angle between the femoral posterior condylar line and the tibial ML axis. Angles are positive for external rotation of the tibia relative to the femur (same convention as the standard TFR method).

Segmental tibial torsion was measured on CT as previously described (angle between the posterior tibial condylar axis proximally and the bimalleolar axis distally).

All primary TFR measurements were performed by the first author experienced in PF imaging, using the measurement definitions described above. To assess intra‐rater reliability, the same observer repeated the measurements in a random sample of 10 knees in a separate session, blinded to the initial measurements. To assess inter‐rater reliability, a senior author, with expertise in PF imaging, independently measured the same 10 cases, blinded to the primary observer's measurements. To minimize expectation bias, CT and MRI measurements were performed in separate sessions, and the observer was blinded to the values obtained on the other modality at the time of measurement.

### Statistical analysis

Given the retrospective design and the fixed number of eligible cases with paired CT and MRI, an a priori sample size calculation was not conducted. A paired samples *t* test was conducted to evaluate systematic inter‐modality mean differences between CT and both standard MRI method and plateau‐anchored MRI method. A *p* < 0.05 was considered statistically significant (IBM SPSS v15.0). Inter‐ and intra‐rater reliability was assessed using Cohen's weighted *κ* on measurements on a random sample of 10 cases. Bland‐Altman plots were constructed to graphically represent these findings. Exploratory subgroup analyses were performed to evaluate whether the mean CT–MRI TFR differences varied by presenting diagnosis (PF instability ± pain vs. pain without PF instability). For subgroup analyses, CT–MRI mean differences were compared between indications using standard parametric testing for continuous outcomes (two‐sided *p* < 0.05). Association between tibial torsion and CT‐measured TFR was evaluated using Pearson correlation [6, 12, 17, 21].

## RESULTS

Fifty‐eight knees from 46 patients were included in the present retrospective study. Fifty knees from females and eight knees from males were included. The average age at the time of surgery was 21 ± 7 years. The average body mass index (BMI) registered prior to surgery was 25 ± 6. The reason for surgery was PF instability ± pain in 20 knees and pain without PF instability in 38 knees.

The mean value of the TFR angle measured on MRI and CT scan was 2.11° and 8.28°, respectively. The mean angle was 9.80° for the alternate proposed measurement for TFR on MRI. The mean difference between MRI and CT for the standard method was −6.18° (95% confidence interval [CI]: −7.48 to −4.88, *p* < 0.001). In contrast, the mean difference between the alternate method and CT was 1.51° (95% CI: 0.01–3.01, *p* = 0.048).

**Figure 4:**
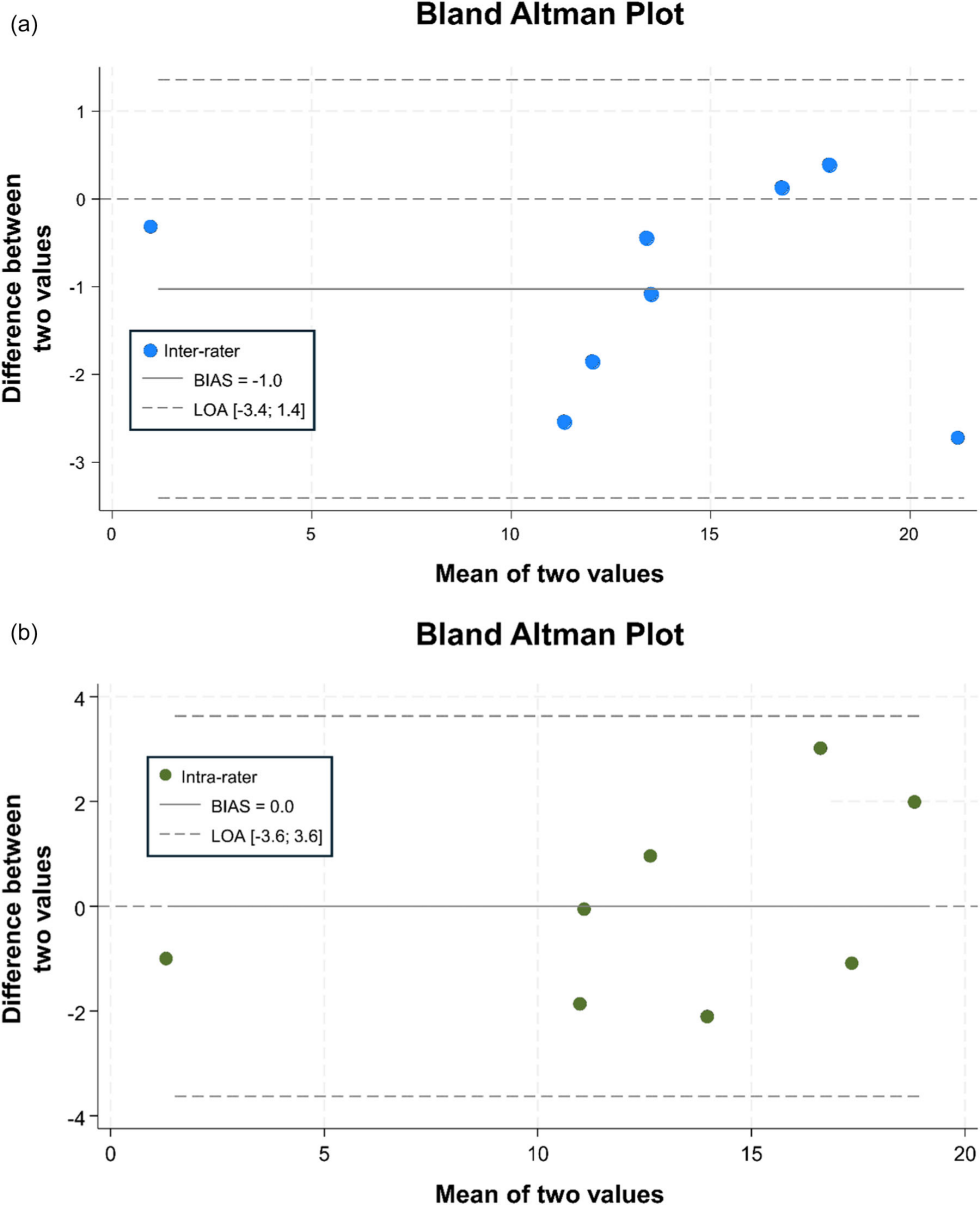
Bland‐Altman plot of inter‐class (a) and intra‐class (b) measurement agreement. Limits of agreement (LOA) are represented by the dotted lines, and the solid line represents the bias.

Intra‐rate reliability on a random selection of 10 cases resulted in a *κ* score of 0.629 with a *p* value of 0.003 (95% CI 0.448–0.810). Inter‐rater reliability on the same set of 10 cases resulted in a *κ* score of 0.722 with a *p* value of <0.001 (95% CI 0.594–0.849). Bland‐Altman plots of inter‐class correlation (Figure 4a) and intra‐class correlation (Figure 4b) showed good agreement between these measurements.

No statistically significant differences were observed in exploratory comparisons of CT–MRI mean differences between indications (PF instability ± pain vs. pain without PF instability).

**Figure 5:**
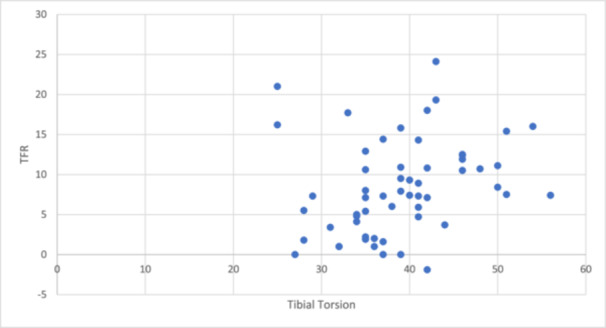
Scatterplot of tibial torsion (°) versus TFR (°) measured on CT. Each dot is one knee. No meaningful correlation was observed (Pearson's *r* = 0.25, *p* = 0.06). CT, computed tomography; TFR, tibiofemoral rotation.

Measured tibial torsion was 38.90° ± 6.99°. When tibial torsion was plotted against the TFR measure on CT scans, there was no statistically significant correlation found (Pearson's *r* = 0.25, *p *= 0.06, Figure 5).

## DISCUSSION

The main finding of the present study is that MRI and CT produce different TFR measurements in the same knees, with MRI values lower than CT in this cohort. A secondary finding is that a plateau‐anchored MRI method yielded a smaller mean difference relative to CT than the standard MRI method. In exploratory subgroup analyses based on surgical indication (PF instability ± pain vs. pain without PF instability), it was not observed that there were statistically significant differences in the CT–MRI mean TFR differences between subgroups. There was no association between the CT‐based TFR measurement and CT‐based tibial torsion measurements.

These results help interpret a growing literature that links through‐knee rotation to PF pathomechanics. On MRI, TFR increases stepwise with clinical severity of PF instability—highest in fixed/obligatory dislocators, intermediate in standard traumatic instability, and lowest (slight internal rotation) in controls—with excellent inter‐rater reliability (intraclass correlation coefficient [ICC] ≈ 0.96) [11]. Moreover, TFR correlates (moderately to strongly) with tibial tubercle–trochlear groove (TT–TG) in the more severe cohorts, underscoring rotation–translation coupling at the knee [11]. On CT, greater TFR correlates with worse patellar maltracking metrics, such as patellar tilt, and with clinical phenotypes of RPD [24].

CT and MRI may differ, for TFR, for technical and biologic reasons that make it difficult to identify a gold standard. CT typically offers isotropic voxels and sharp cortical edges, reducing landmark ambiguity. MRI relies on discrete 2D slices, where slice level, partial‐volume effects, fat‐sat/susceptibility, and cartilage contour visibility can shift the apparent axes. Differences in knee extension, leg support, coil orientation, quadriceps relaxation, and foot rotation during acquisition can also change the final TFR measured. Finally, anchoring the tibial axis to the posterior condylar line (standard MRI) versus the plateau ML axis (alternate MRI) samples different anatomic references at the joint level, possibly due to the different slice thickness of the imaging modality. Together, these factors plausibly yield lower MRI values relative to CT in this cohort, without asserting that either modality is the reference standard.

These findings complement those of Jud et al., who reported systematic differences between CT‐ and MRI‐derived TFR in patients with PF instability. In contrast, in this study, a broader surgical PF cohort (including pain without instability) was evaluated and was introduced a plateau‐anchored MRI reference aimed at reducing the mean CT–MRI difference observed with the standard posterior tibial reference [8].

This novel understanding of TFR on CT vs. MRI could impact clinical practice and surgical decision‐making. Most PF clinicians routinely obtain MRI to characterize anatomic risk factors. However, previous works showed how the TFR inflates TT–TG even without true tubercle lateralization [1, 14, 15, 24], making the TT–TG appearing more dependent on rotation itself than tubercle lateralization, in RPD populations [3, 23]. This coupling means that small inter‐slice or inter‐plane mismatches across modalities can amplify apparent differences. Hence, decisions based solely on TT–TG thresholds can misclassify patients if rotation is unaccounted for. Since standard MRI TFR ≠ CT TFR, MRI may underestimate true rotational malalignment. While the primary message of this study is that CT and MRI differ for TFR, a practical secondary takeaway is that a plateau‐anchored MRI measurement may direct future research to a pragmatic MRI‐based alternative, which showed a smaller mean difference relative to CT than the standard MRI method in this cohort; however, this finding should be interpreted as hypothesis‐generating rather than demonstrating CT‐equivalence or accuracy. If these exploratory findings will be validated, when TT–TG is high but TT–PCL is normal or borderline, obtaining CT or a plateau‐anchored MRI method TFR measurement before recommending tubercle medialization could be useful, to avoid addressing a rotation‐driven problem with a translation‐only solution.

The present study has several limitations. This retrospective series was comprised of surgical candidates only, which may limit generalizability to a broader subset of knee conditions. The cohort was predominantly female, which may limit generalizability and could influence measured rotational values. Knee flexion angle at acquisition was not assessed in this retrospective dataset and represents a major source of potential bias, as positioning differences may influence measured TFR and preclude mechanistic interpretation of CT–MRI differences. Furthermore, MRI scans were obtained on heterogeneous scanners or protocols outside a controlled research protocol; while this reflects real‐world practice, it introduces variability in slice angulation, resolution, and landmark depiction that could accentuate inter‐modality differences. Indeed, MRI‐generated images did not have a standardized coil type, knee flexion/extension, or foot rotation at acquisition, which are potential sources of systematic inter‐modality differences. Additionally, the CT and MRI were not performed on the same day. TFR measurements can be sensitive to landmark selection and slice choice, and observer‐dependent variability has been reported for rotational measurements. Therefore, these findings should be interpreted as describing systematic mean differences between modalities in this cohort rather than establishing reproducibility of the measurements or interchangeability between CT and MRI. Future studies should incorporate blinded repeated measurements by multiple observers to quantify measurement reliability. Finally, TFR values were not linked to postoperative outcomes.

Further prospective studies should aim to standardize MRI positioning and acquisition parameters (i.e., cadaveric study), include same‐day CT comparators to isolate modality effects, report reliability for plateau‐anchored MRI TFR, and correlate modality‐specific TFR with surgical indications and outcomes (e.g., derotational osteotomy vs tubercle transfer) to establish actionable thresholds. Given the rotation–translation coupling, future work should also model TFR‐adjusted TT–TG in risk stratification and decision algorithms.

## CONCLUSIONS

CT and MRI yield systematically different TFR values in the same PF patients, with standard MRI yielding lower values than CT. A plateau‐anchored MRI method yielded a smaller mean difference relative to CT than the standard MRI method in this cohort. These findings should not be interpreted as demonstrating interchangeability or accuracy of MRI relative to CT neither suggest clinical adoption of a plateau‐anchored MRI method; rather, they support further prospective work to develop and validate an MRI‐centred approach to rotational assessment and surgical decision‐making.

## AUTHOR CONTRIBUTIONS

*Study conception and design*: Giuseppe Anzilotti and Elizabeth A. Arendt. *Material preparation*: Giuseppe Anzilotti, Elizabeth A. Arendt and Marc A. Tompkins. *Data collection*: Spencer M. Comfort and Giuseppe Anzilotti. *Analysis*: Julie Agel. The first draft of the manuscript was written by Giuseppe Anzilotti. All authors read and edited versions of the manuscript. All authors approved the final manuscript.

## CONFLICT OF INTEREST STATEMENT

The authors declare no conflicts of interest.

## ETHICS STATEMENT

This study was approved by the University of Minnesota IRB Study 00020032.

## Data Availability

Available upon request from the corresponding author.
